# A basic ddRADseq two‐enzyme protocol performs well with herbarium and silica‐dried tissues across four genera

**DOI:** 10.1002/aps3.11344

**Published:** 2020-04-23

**Authors:** Ingrid E. Jordon‐Thaden, James B. Beck, Catherine A. Rushworth, Michael D. Windham, Nicolas Diaz, Jason T. Cantley, Christopher T. Martine, Carl J. Rothfels

**Affiliations:** ^1^ University Herbaria and Department of Integrative Biology University of California Berkeley 3040 Valley Life Sciences Building Berkeley California 94720 USA; ^2^ Department of Biological Sciences Wichita State University 1845 Fairmount Wichita Kansas 67260 USA; ^3^ Botanical Research Institute of Texas 1700 University Drive Fort Worth Texas 76107 USA; ^4^ Department of Evolution and Ecology and Center for Population Biology University of California Davis One Shields Avenue Davis California 95616 USA; ^5^ Department of Plant and Microbial Biology University of Minnesota 1500 Gortner Avenue St. Paul Minnesota 55108 USA; ^6^ Department of Biology Duke University 130 Science Drive Durham North Carolina 27708 USA; ^7^ Department of Biology Bucknell University 1 Dent Drive Lewisburg Pennsylvania 17837 USA; ^8^ Present address: Department of Botany University of Wisconsin 430 Lincoln Drive Madison Wisconsin 53706 USA; ^9^ Present address: Biology Department Portland State University 1719 SW 10th Avenue Portland Oregon 97201 USA; ^10^ Present address: Department of Biology San Francisco State University 1600 Holloway Avenue San Francisco California 94132 USA

**Keywords:** *Boechera*, double‐digest restriction site–associated DNA sequencing (ddRADseq), *Draba*, herbarium specimens, *Ilex*, *Solidago*

## Abstract

**Premise:**

The ability to sequence genome‐scale data from herbarium specimens would allow for the economical development of data sets with broad taxonomic and geographic sampling that would otherwise not be possible. Here, we evaluate the utility of a basic double‐digest restriction site–associated DNA sequencing (ddRADseq) protocol using DNAs from four genera extracted from both silica‐dried and herbarium tissue.

**Methods:**

DNAs from *Draba*,* Boechera*,* Solidago*, and *Ilex* were processed with a ddRADseq protocol. The effects of DNA degradation, taxon, and specimen age were assessed.

**Results:**

Although taxon, preservation method, and specimen age affected data recovery, large phylogenetically informative data sets were obtained from the majority of samples.

**Discussion:**

These results suggest that herbarium samples can be incorporated into ddRADseq project designs, and that specimen age can be used as a rapid on‐site guide for sample choice. The detailed protocol we provide will allow users to pursue herbarium‐based ddRADseq projects that minimize the expenses associated with fieldwork and sample evaluation.

Genomic tools that best combine data quality, ease, and cost‐effectiveness become standard in the empirical studies that advance our knowledge of diversity and phylogeny. Of the six broad categories of genomic tools outlined by McKain et al. (2018), restriction site–associated DNA sequencing (RADseq) and target enrichment approaches most effectively combine the potential for generating large data sets with applicability to samples of varying DNA quality. Although herbarium‐derived DNAs (“herbarium DNA”) are now being included in both target enrichment (Hart et al., 2016; Brewer et al., 2019) and RADseq (see references below) studies, concerns remain regarding the link between data recovery and herbarium DNA degradation. Here, we define herbarium DNA degradation as comprising any type of alteration that occurs during collection, processing, and museum storage that negatively affects DNA extract quality. The most recognized form of DNA degradation are double‐strand breaks, which we will refer to as DNA shearing. DNA shearing is viewed as particularly problematic for RADseq, because highly sheared DNAs could include relatively few intact fragments flanked by appropriate cut sites (Graham et al., 2015). Graham et al. (2015) found a negative relationship between DNA shearing and RADseq data recovery in freshly collected fish tissue, and data loss was significantly more severe at the highest level of shearing. Beck and Semple (2015) similarly reported a strong relationship between data loss and herbarium specimen age when using genotyping‐by‐sequencing, but relied on only a coarse, agarose gel–based visual assessment of shearing. Other studies reporting RADseq success with herbarium specimens did not include a formal evaluation of specimen age or DNA degradation on the levels of RADseq data recovery (Massatti et al., 2016; Wessinger et al., 2016; Gilman and Tank, 2018).

A broader evaluation of the feasibility of RADseq with herbarium DNA is needed. In this study, we assess the relationships between preservation method, specimen age, DNA shearing, and data recovery both within and across four sample sets representing three angiosperm families, four genera, and both silica‐dried and herbarium tissues. A double‐digest RADseq (ddRADseq) protocol was used to process all samples. The success of a single, streamlined protocol incorporating a single restriction enzyme pair would reduce upfront enzyme costs and allow for the simultaneous preparation of ddRADseq libraries from diverse sample sets. This flexibility would allow a researcher to quickly assemble geographically and taxonomically broad sample sets by utilizing herbarium DNAs, thus reducing the need for costly fieldwork and allowing for sampling regimes that would otherwise be unattainable.

## METHODS

### Sampling and DNA extraction, desalting, and assessment

A detailed bench protocol is presented in Appendix S1, and descriptive data for the 192 samples are included in Appendix S2. The 48‐sample, 44‐taxon *Draba* L. (Brassicaceae) pool comprised 20 silica‐dried and 28 herbarium samples extracted using a standard cetyltrimethylammonium bromide (CTAB) protocol (Doyle and Dickson, 1987). The 48‐sample, 13‐taxon *Boechera* Á. Löve & D. Löve (Brassicaceae) pool comprised eight silica‐dried and 40 herbarium samples extracted using the 96‐well CTAB protocol outlined in Beck et al. (2012). The 48‐sample, 19‐taxon *Solidago* L. (Asteraceae) pool comprised 48 herbarium samples extracted using the 96‐well CTAB protocol with the addition of 400 μg of RNase A (QIAGEN, Hilden, Germany) during incubation. The 48‐sample, single‐taxon *Ilex* L. (Aquifoliaceae) pool comprised 48 silica‐dried samples extracted using the standard CTAB protocol. All DNAs that did not undergo a standard RNase treatment during the extraction were incubated (post‐extraction) at 37°C for 1 h with 100–200 μg of RNase A (QIAGEN). All extracts were desalted (see Appendix S1).

DNA degradation was quantified as the DNA integrity number (DIN) for the *Draba*,* Boechera*, and *Solidago* samples, which was determined using a Genomic DNA ScreenTape Assay on a TapeStation 2200 (Agilent Technologies, Santa Clara, California, USA). *Ilex* DIN values were not determined for logistical reasons. DIN values range from 1–10, with lower numbers indicating more degradation. Genome sizes estimated from C‐values (C‐value database; Leitch et al., 2019) included 0.23 Gbp (*Boechera*), 0.39 Gbp (*Draba*), and 1.1 Gbp (*Solidago* and *Ilex*). While the *Draba* data set included both diploids and polyploids, all *Boechera*,* Solidago*, and *Ilex* individuals were diploid.

### Enzyme choice, library preparation, sequencing, and single‐nucleotide polymorphism (SNP) calling

Prior success with *Eco*RI*/Sph*I in *Draba* (Jordon‐Thaden, University of California Berkeley, unpublished data) suggested that this enzyme pair would also be suitable for the confamilial *Boechera*. In silico digests of the *Daucus carota* L. genomic sequence in Geneious version 10.2 (Biomatters Ltd., Auckland, New Zealand; see Appendix S1) suggested that the *Eco*RI/*Sph*I enzyme pair would produce the target number of fragments in the *Solidago* and *Ilex* genomes as well. Library preparation involved a modified version of the protocol from Peterson et al. (2012), and followed the detailed protocol presented in Appendix S1. A significant cost‐saving measure of this protocol is the “freeze and squeeze” size selection approach during library preparation (Appendix S1, section I). This technique eliminates the need for a digital size selection apparatus (BluePippin; Sage Science, Beverly, Massachusetts, USA) in the user lab. Rather, fragment analysis (TapeStation) and size selection of final libraries (if needed) can be performed at the chosen sequencing facility for a modest fee.

The *Boechera*/*Draba* and *Solidago*/*Ilex* pools were combined, and each pool pair was sequenced with 150‐bp paired‐end sequencing on separate HiSeq 2500 lanes (Illumina, San Diego, California, USA) at the University of Kansas Genome Sequencing Core. Demultiplexing, clustering, and SNP calling were conducted for each 48‐sample pool using PyRAD version 3.0 (Eaton, 2014). The PyRAD settings for each of the data runs were as follows. In step 1, the restriction overhangs CATG and AATT (*Eco*RI and *Sph*I) were specified, with data type “pairddrad.” Runs were performed on the SAVIO server of the University of California Berkeley Computing Facility (one node with 20 tasks per node running in parallel), allowing zero barcode mismatches during demultiplexing. Before continuing with PyRAD, PEAR (Zhang et al., 2014) was used to assemble paired reads. All non‐assembled reads were discarded, and the assembled reads were used in all downstream PyRAD analyses. Each taxon‐specific pool was then processed with steps 2–7 in PyRAD, using vsearch and muscle to create the multiple sequence alignments of the assembled reads, using the following parameters: 24 parallel processors, minimum cluster sequence coverage at 6, the maximum number of sites with qualifiers less than 20 at 4, the clustering threshold at 85%, data type as ddrad, maximum number of shared polymorphic sites in a locus at 3, and maximum number of heterozygosity sites in the consensus sequences at 10. All other parameters were left at default values. Separate runs with the above parameters, but with differing minimum taxon coverage thresholds (the minimum number of samples represented in a locus for that locus to be included: min_samples_locus = 4, 6, 12, and 24), were conducted for each taxon pool. Following these initial runs, low‐yield samples were removed (<10% of the pool mean assembled reads or from which <15% of the total reads were assembled) and PyRAD runs at each minimum taxon coverage threshold were performed for each data set in order to generate data sets for phylogeny construction.

### Statistical and phylogenetic analyses

We first explored which variable to use as an assessment of output quality: the number of eventual loci available for tree construction, or the number of assembled reads. Because ddRADseq across divergent taxa is subject to locus dropout, which can have substantial impacts on the number of loci available for tree construction, we used the number of assembled reads as an estimate of ddRADseq success. Regardless, we note that the number of assembled reads is positively correlated with the number of downstream loci (“pyrad_N_4_nloci,” Appendix S2) (Pearson's product moment correlation = 0.91, *t* = 30.84 on 190 df, *P* < 0.0001). We first normalized the number of assembled reads (divided the number for a given sample by the average number of assembled reads for all samples in the genus data set) and then log‐transformed it, which improved model fit as estimated by visually assessing Pearson residuals. Silica‐dried samples were excluded from all analyses incorporating age, and herbarium sample age was centered and scaled using the “scale” function.

We performed correlation tests and linear models in R version 3.5.1 (R Core Team, 2018). We first modeled ddRAD success for the full 192‐sample data set as a function of taxon and preservation type (silica dried vs. herbarium tissue), after removing three samples determined to be outliers from a visual assessment of the residuals (*Draba* sample UC37 and *Ilex* samples RR_4U_N119 and RS_11F_N166). In the second linear model, we asked whether the effects of herbarium sample age varied by taxon, coded as an interaction term. For this herbarium‐only analysis, we removed the *Ilex* samples (all of which were silica dried), eight silica‐dried *Boechera* samples, 20 silica‐dried *Draba* samples, and three outliers (*Draba* UC37, and *Solidago* samples JB2586 and JB2587). We note that because taxon and sequencing lane are confounded, models accounting for variation due to lane were not estimable. It is likely that some variation attributed to taxon is due to variation in lane. To assess significance, we used the ANOVA function from the car package (Fox et al., 2019) to calculate the Type II sums of squares for the entire data set model, and Type III sums of squares for the herbarium‐only data set.

All phylogenetic analyses were performed with low‐yield samples removed (<10% of the pooled mean assembled reads or from which <15% of the total reads were assembled). For each genus, maximum likelihood (ML) phylogenies and bootstrap searches were performed on concatenated locus alignments resulting from each of four PyRAD filtering thresholds: min_samples_locus = 4, 6, 12, and 24. ML phylogenies were inferred from the unpartitioned data using Garli version 2.0.1019 (Zwickl, 2006), under the default settings (except that “availablememory” was increased to 3500), on the CIPRES gateway (Miller et al., 2010), with support assessed via 600 bootstrap pseudoreplicates. All analyses used a GTR+I+G substitution model. The searches for the ML trees were performed from two independent random‐addition starting trees and the bootstrap searches were each performed once, from a single random‐addition starting tree. The ML trees for each analysis were annotated with their bootstrap support values using sumtrees version 4.4.0 in the DendroPy version 4.4.0 package (Sukumaran and Holder, 2010, 2018), and support values were summarized and compared with custom R scripts (R Core Team, 2018) using the ape, ggplot2, and phanghorn packages (Paradis et al., 2004; Schliep, 2011; Wickham, 2016).

It should be noted that this study is intended as a proof of concept of the use of ddRADseq with herbarium material, and our phylogenetic conclusions themselves should be interpreted with caution. Specifically, our ML analyses assume that the sites in an alignment are homologous to each other—that is, that they share a single evolutionary history. Given that the great majority of our ddRADseq markers are from the nucleus, this assumption will be frequently violated in our data sets that have extensive intraspecific sampling, where independent assortment and recombination will result in individual markers with distinct evolutionary histories (Moore, 1995). Researchers interested in using ddRADseq data to infer relationships within species should instead utilize methods that do not rely on concatenation (e.g., polymorphism‐aware phylogenetic models [PoMo]; Schrempf et al., 2016).

## RESULTS

### DNA quantity and quality, sequencing success, and SNP recovery

Full details regarding sample DNAs and downstream data (raw read number, loci recovered, etc.) are presented in Appendix S2 (both Appendix S2 and the values reported in this section reflect all 192 samples). The DNA concentrations following desalting ranged from 12–110 ng/μL (mean 59.0 ± 26.9 ng/μL) in the *Draba* pool, 20.1–92.9 ng/μL (mean 47.2 ± 17.5 ng/μL) in the *Boechera* pool, 19.1–84.4 ng/μL (mean 59.0 ± 14.7 ng/μL) in the *Solidago* pool, and 2–164 ng/μL (mean 33.4 ± 33.8 ng/μL) in the *Ilex* pool. It should be noted that we did not fully normalize DNA input due to the time needed to add a custom volume for each of the 192 samples during the time‐sensitive double‐digest preparation (Appendix S1, section E.2). We did, however, attempt to add similar amounts of starting DNA. Because the optimal per‐sample input DNA is 0.3 μg and the recommended per‐sample DNA input volume is 5 μL, 60 ng/μL is the optimal input DNA concentration. We targeted this optimal DNA concentration in our pre‐digest desalting step (Appendix S1, section C). DNA input to the initial double digest varied from 0.01–0.8 μg (0.248 ± 0.132).

**Figure 1:**
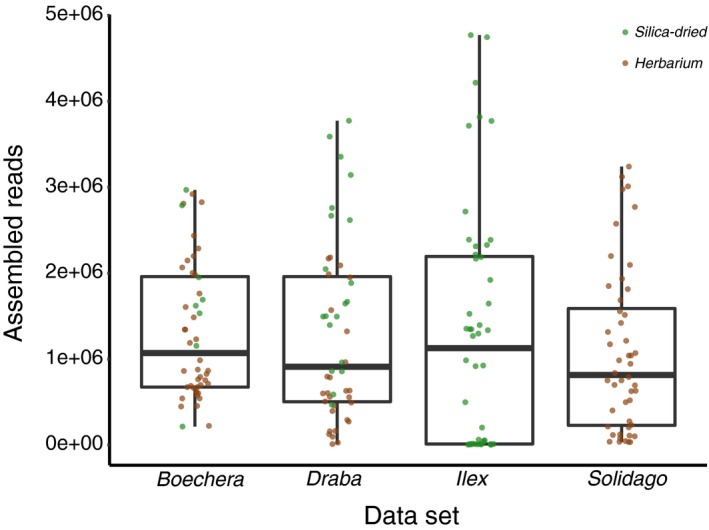
Assembled reads (raw values) recovered for all 192 samples, organized by data set and tissue type. Boxes illustrate medians and interquartile ranges.

TapeStation DIN values were obtained for 47 of the 48 *Draba* individuals, 44 of the 48 *Boechera* individuals, and all 48 *Solidago* individuals (Appendix S2). These values were 1.0–7.1 (mean 3.0 ± 2.1) in the *Draba* pool, 1.0–7.2 (mean 5.0 ± 1.5) in the *Boechera* pool, and 1.0–6.9 (mean 3.4 ± 1.6) in the *Solidago* pool (Appendix S2). The number of assembled reads per sample was 11,397–3,774,022 (mean 1,274,849 ± 1,010,248) in the *Draba* pool, 214,676–2,968,827 (mean 1,313,263 ± 792,928) in the *Boechera* pool, 34,516–3,240,725 (mean 1,071,172 ± 941,066) in the *Solidago* pool, and 3,716–4,770,722 (mean 1,292,866 ± 1,411,310) in the *Ilex* pool (Fig. 1, Appendix S2). The number of loci recovered per sample at the min_samples_locus = 4 setting (a locus has to be present in at least four samples to be included) was 8–3333 (mean 1680 ± 972) in the *Draba* pool, 479–2618 (mean 1132 ± 463) in the *Boechera* pool, 178–6527 (mean 2186 ± 1568) in the *Solidago* pool, and 7–10,117 (mean 2430 ± 2557) in the *Ilex* pool (Appendix S2).

### Factors affecting data recovery

**Figure 2:**
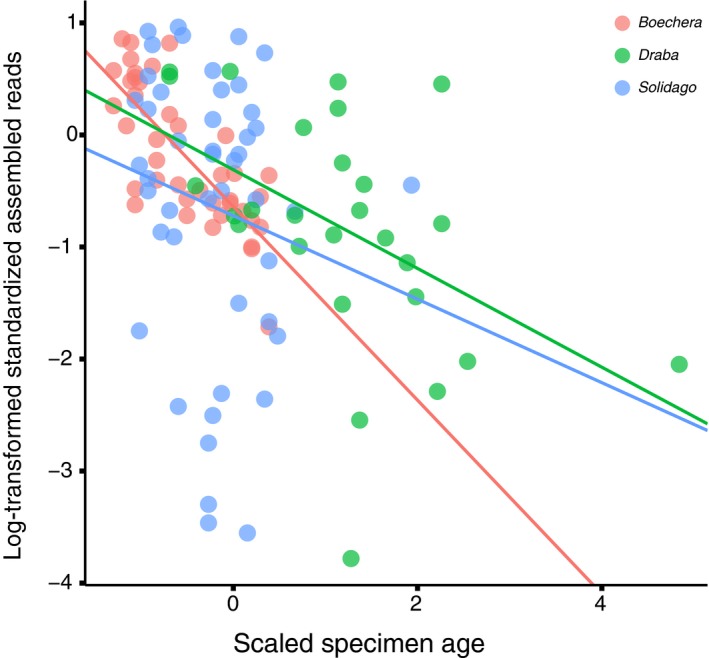
The number of log‐transformed normalized assembled reads is negatively correlated with herbarium specimen age (scaled and centered). Lines for each taxon represent the linear regression output (Table 2).

In the full 192‐sample data set, log‐transformed normalized assembled reads were positively correlated with DIN (Spearman's *rho* = 0.39, *S* = 274680, *P* < 0.0001). Higher DIN values indicate less DNA shearing; thus DNA that was more intact was associated with a higher read number. This was similarly reflected in the herbarium‐only data set (Spearman's *rho* = 0.35, *S* = 152870, *P* = 1.77e–04). Log‐transformed normalized assembled reads were negatively correlated with herbarium specimen age (Pearson's product moment correlation = −0.32, *t* = −3.67 on 114 df, *P* = 3.76e–04), with older specimens associated with lower read numbers. Linear modeling of the full 192‐sample data set as a function of taxon and preservation mode revealed that both factors significantly contributed to the variation in the assembled read number (adjusted *R* ^2^ = 0.1, *F*_(4,184)_ = 6.41, *P* < 0.0001; Table 1). Analysis of the herbarium‐only data set showed that specimen age contributed to assembled read number, with older specimens producing fewer successfully assembled reads (adjusted *R* ^2^ = 0.17, *F*_(5,107)_ = 5.57, *P* = 1.33e–04; Table 2). Neither taxon nor the taxon*age interaction significantly contributed to read variation. The slope of reads vs. age does differ among taxa (Fig. 2), although not significantly so (Table 2).

**Table 1:** Results from a linear model of log‐transformed normalized assembled reads as a function of taxon and preservation mode (“Preserve,” silica‐dried vs. herbarium tissue).^a^

	Level	Estimate	SE	SS	df	*F*	*P* value
**Intercept**	—	−0.27	0.23	—	—	—	—
**Taxon**	*Draba*	−0.43	0.32	56.09	3	8.23	<0.0001
	*Ilex*	−1.99	0.43				
	*Solidago*	−0.48	0.31				
**Preserve**	Silica	0.83	0.35	12.49	1	5.5	0.02

^a^Coefficients for each level of predictor variables are provided from the model summary. The significance for each variable was assessed using Type II sums of squares (SS). One taxon is represented as the reference category in the model, and thus does not appear as an effect.

**Table 2:** Results from a linear model of log‐transformed normalized assembled reads as a function of taxon and specimen age (herbarium specimens only).^a^

	Level	Estimate	SE	SS	df	*F*	*P* value
**Intercept**	—	−0.64	0.21	8.53	1	9.44	2.69e‐03
**Taxon**	*Draba*	0.33	0.33	1.68	2	0.93	0.40
	*Solidago*	−0.08	0.26				
**Age**	—	−0.86	0.28	8.78	1	9.72	2.34e‐03
Taxon*Age	Draba*age	0.42	0.32	1.93	2	1.07	0.35
	Solidago*age	0.49	0.37				

a Coefficients for each level of predictor variables are provided from the model summary. The significance for each variable was assessed using Type III sums of squares (SS). One taxon is represented as the reference category in the model, and thus does not appear as an effect.

### Phylogenetic utility of ddRADseq data sets

Four samples were removed from the *Draba* pool due to low numbers of assembled reads, and 0, 8, and 19 samples were similarly removed from the *Boechera*,* Solidago*, and *Ilex* pools, respectively. Average bootstrap support was highest at the min_samples_locus = 4 threshold for all four genera (data not shown). Maximum likelihood trees inferred from these four data sets are shown in Appendices [Link], [Link], [Link], [Link] and are discussed briefly here. The intraspecific *Ilex opaca* Aiton data set exhibited the lowest average bootstrap support at (0.513), although a number of highly supported (>90% bootstrap) internal branches suggest that individuals from putatively natural populations of this species are frequently most closely related to individuals from the same natural population as opposed to those from likely planted populations (Appendix S3). The *Draba* data set exhibited the third highest average bootstrap support (0.756) and exhibited highly supported branches, most notably along the backbone (Appendix S4). The data set recovered previously observed relationships among the genera *Tomostima* Raf., *Abdra* Greene, and *Draba*, and the three primary *Draba* geographic clades (Jordon‐Thaden et al., 2010). The *Solidago* data set exhibited the second highest average bootstrap support (0.798), and highly supported clades corresponded to relationships at both shallow (10 of 14 species or subspecies were monophyletic) and deeper levels (*Solidago* series *Odorae* (Mack.) Semple and *Solidago* subsection *Triplinerviae* (Torr. & A. Gray) G. L. Nesom were monophyletic; Appendix S5). The *Boechera* data set exhibited the highest average bootstrap support (0.957)—only two internal nodes did not receive maximum ML bootstrap support (Appendix S6). The monophyly of the genus and of all 11 included *Boechera* species are strongly supported.

## DISCUSSION

### ddRADseq utility across taxa and tissue quality

**Figure 3:**
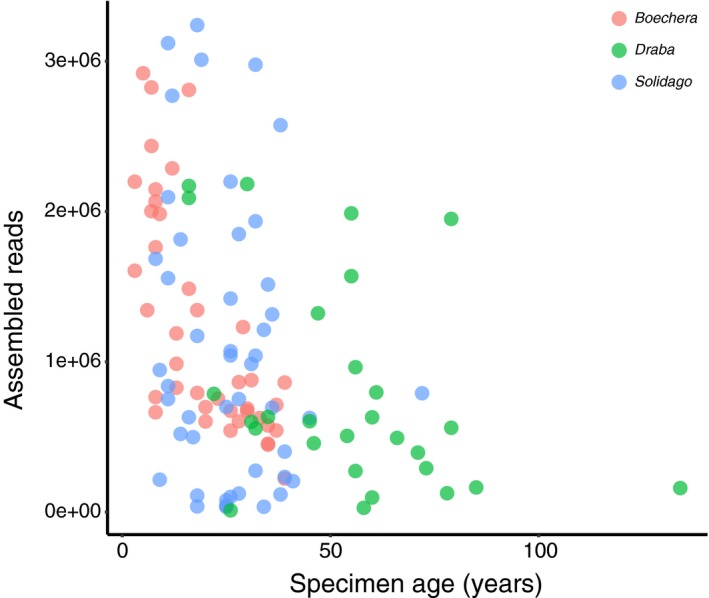
The relationship between the obtained assembled reads (raw values) and herbarium specimen age (raw values) in the three genera for which herbarium specimens were sampled.

Although taxon, preservation method, and herbarium specimen age each significantly impacted the number of reads recovered (Tables 1, 2), these effects were not overwhelming. Many samples (139 of 192, 72%) from all four genera recovered >500,000 reads, including many herbarium samples (86 of 116, 74%; Fig. 1). The largest variation in success was observed in the silica‐dried‐only *Ilex* sample set (Fig. 1), suggesting that specifics of plant chemistry and structure (leaf thickness; see Neubig et al., 2014) could impact success more significantly than tissue degradation during herbarium storage. Indeed, DNA extractions from *Ilex opaca* were found to be visually inconsistent in both pellet size and color. This is perhaps due to its thick cuticle and the fact that material was collected in the winter in order to more easily locate individuals. The observation that many DNAs derived from herbarium specimens perform similarly to those derived from silica‐dried tissue suggests that, instead of a last resort for rare or hard‐to‐collect taxa, herbarium specimens should be viewed as equally viable sampling options. Although Särkinen et al. (2012) reported that silica‐dried tissues were associated with higher single‐gene Sanger sequencing success, this was most evident with longer amplicons. ddRADseq typically targets regions less than 400 bp, likely diminishing this effect. We observed that herbarium specimen age had a notable effect on ddRADseq data recovery, and note that Brewer et al. (2019) found specimen age to be negatively correlated with target enrichment success. Taken together, these results highlight age as an instant, straightforward, cost‐free metric for choosing among otherwise geographically and morphologically equivalent specimens (Fig. 3). Herbarium specimen age was strongly negatively correlated with DIN (Spearman's *rho* = –0.73, *S* = 415030, *P* < 0.0001), with older specimens exhibiting lower DIN values (higher degree of shearing). Brewer et al. (2019) also observed a higher degree of shearing in older herbarium DNAs. Although the effect of age could therefore be largely a function of shearing itself, the role of additional age‐related effects beyond simple strand breaks should be investigated (Staats et al., 2011; Weiß et al., 2016). Regardless, specimen choice using age alone is preferable to the expensive and labor‐intensive process of sampling, extracting, and assessing DNA shearing via fragment analysis or gel electrophoresis in large numbers of candidate specimens.

### ddRADseq with herbarium specimens: Prospects and further study

It is important to note that these results were obtained from a narrow phylogenetic, physical, and geographic context, and that the observed success with herbarium specimens and the effect of specimen age will likely not extend to all scenarios. Our samples represent only three angiosperm families, are of relatively thinned‐leaved taxa, and herbarium specimens were obtained exclusively from temperate herbaria. Neubig et al. (2014) suggested that leaf thickness and clade‐specific effects could influence DNA shearing, and both Neubig et al. and others have reported evidence of collection and curatorial practice effects on DNA shearing and/or PCR success (Ribeiro and Lovato, 2007; Adams, 2011). Specimens collected in temperate regions are generally air‐dried (with or without heat), while specimens collected in tropical regions are often first collected in alcohol (i.e., the Schweinfurth method) (Schrenk, 1888). Herbaria also archive specimens using a broad range of mounting, temperature, humidity, and pest control protocols (Neubig et al., 2014). These uncertainties aside, we feel that the results of this study should encourage researchers to aggressively pursue ddRADseq methods in conjunction with the massive trove of geographically, temporally, and morphologically explicit plant specimens housed and curated in the world's herbaria (Heberling and Isaac, 2017; James et al., 2018).

## Supporting information


**APPENDIX S1.** ddRADseq protocol modified from Peterson et al. (2012).

**APPENDIX S2.** Sample information (taxon, year collected, herbarium of origin, DIN) and ddRADseq results (e.g., read and loci number).

**APPENDIX S3.** Unrooted phylogeny of 29 *Ilex opaca* samples using a data set filtered at the threshold min_samples_locus = 4.

**APPENDIX S4.** Phylogeny of 43 *Draba* (or outgroup) samples using a data set filtered at the threshold min_samples_locus = 4.

**APPENDIX S5.** Unrooted phylogeny of 40 *Solidago* samples using a data set filtered at the threshold min_samples_locus = 4.

**APPENDIX S6.** Phylogeny of 48 *Boechera* samples using a data set filtered at the threshold min_samples_locus = 4.

**APPENDIX S7.** ddRAD library map, with colors corresponding to Appendix S1.

**APPENDIX S8.** Oligo tube order (see Appendix S1).

**APPENDIX S9.** Oligo plate order (see Appendix S1).

## Data Availability

Demultiplexed sequence data and concatenated locus alignments are available on the Dryad Digital Repository (https://doi.org/10.5061/dryad.rbnzs.7h80; Jordon‐Thaden et al., 2020).
