# Supplementary material for: A basic ddRADseq two‐enzyme protocol performs well with herbarium and silica‐dried tissues across four genera
Source: Appl Plant Sci. 2020 Apr 23;8(4):e11344. doi: 10.1002/aps3.11344 (PMC7186894; doi:10.1002/aps3.11344)
Supplement: Supplementary file 1 — APPENDIX S1. ddRADseq protocol modified from Peterson et al. (2012). [file APS3-8-e11344-s001.pdf]

DOI

APPENDIX S1. ddRADseq protocol modified from Peterson et al. (2012) by Ingrid Jordon-Thaden (Ingrid.JordonThaden@wisc.edu) and James Beck (james.beck@wichita.edu).

A note on project design. The text below describes work in two 96-well plates (192 samples). These samples comprise four 48-sample "pools." In our case, each pool represented a separate genus. Each pool is a unit, identified by the index added by one of the 12 multiplex PCR primer 2s in the Phusion PCR step. You might prepare more or fewer samples, or only samples from a single species or genus. Regardless, each set of 48 samples will need a unique index so the same set of 48 barcodes (added by the 48 uniquely barcoded P1 adapters) can be used in all pools. Up to 576 individuals (48 barcodes × 12 indices) can therefore be sequenced in one Illumina lane and bioinformatically separated post-sequencing. In this study, however, we only sequenced 96 individuals per Illumina HiSeq 2500 lane. Your sequencing effort will depend on your project design, genome size, desired sequencing coverage, and finances.

A. Choosing the restriction enzyme pair

Use Geneious (Biomatters Ltd., Auckland, New Zealand) to perform "in silico" digests of related taxa with commonly used restriction enzymes. The following steps (Geneious online support, 2014) assume that you are experimenting with the *Eco*RI/*Sph*I enzyme pair.

1. Select your sequence and go to Cloning → Find Restriction Sites. This will open the "Restriction Analysis" tab on the right-hand side of the sequence view.
2. From the dropdown box next to "Candidate enzymes" select "Commonly used enzymes."
3. Click the "Advanced" button.
4. In the dialog box that appears, tick the box next to "Name" to unselect all of the enzymes, then tick the box next to "*Eco*RI," and the box next to "*Sph*I." Click "Save selected enzymes" to save this as a new enzyme set, then click "Done."
5. Untick the box next to "Enzymes must match."
6. Select "Cut anywhere" from the next dropdown box.
7. The restriction enzymes will appear as temporary annotations on your sequence. Click the "Apply" button to apply these annotations to your sequence.
8. Click "Save."

Once you save the sequence, the document view will refresh. Open the new "Fragments" tab to see a list of the length of each fragment. Export this information by clicking "Export table."

By sorting these tables, you can identify the number of "dual-enzyme fragments" in your desired size range. Dual-enzyme fragments are those cut at one end by one of your enzymes and cut at the other end by the other enzyme. Experiment with different enzyme pairs to see which ones provide either too few or too many loci. Too many will result in excess missing data in your final data set, and too few will result in a small number of recovered loci. Ultimately, this choice will depend on the sequencing effort you will apply. Also look for an enzyme pair where one enzyme cuts more frequently (ca. 10,000 cuts), and the other less frequently. The number of cuts that a single enzyme makes can be established by performing additional in silico digests as above, applying one enzyme at a time.

As an example, consider ddRAD preps for goldenrods (*Solidago*) and hollies (*Ilex*). The following values resulted from in silico digests of the first five chromosomes of the carrot (*Daucus carota*) genome, which is in Asterids II along with both *Solidago* and *Ilex*. Each of the chromosome sequence data files were downloaded from the National Center for Biotechnology Information (NCBI; https://www.ncbi.nlm.nih.gov/). Both dual-enzyme (*Eco*RI/*Sph*I) and single-enzyme digests were performed for each chromosome.

chromosome 1 (51 Mbp): 15333/7828 *Eco*RI/*Sph*I cuts; ca. 9% (813 of 9452) of dual-enzyme fragments are 250-450 bp
chromosome 2 (44 Mbp): 12426/6763 *Eco*RI/*Sph*I cuts; ca. 9% (682 of 7952) of dual-enzyme fragments are 250-450 bp
chromosome 3 (50 Mbp): 14340/7563 *Eco*RI/*Sph*I cuts; ca. 9% (823 of 9201) of dual-enzyme fragments are 250-450 bp
chromosome 4 (35 Mbp): 10375/5469 *Eco*RI/*Sph*I cuts; ca. 8% (536 of 6642) of dual-enzyme fragments are 250-450 bp
chromosome 5 (41 Mbp): 11856/6403 *Eco*RI/*Sph*I cuts; ca. 9% (684 of 7599) of dual-enzyme fragments are 250-450 bp

These first five *D. carota* chromosomes = ca. 221 Mbp, in which there are 3538 dual-enzyme fragments between 250–450 bp. Extrapolating to the entire 362 Mbp *D. carota* genome (9 chromosomes) = ca. 5800 such fragments, or ca. 16 “target” fragments per Mbp. Available C-value information (http://data.kew.org/cvalues/) suggests that *Solidago* has a ca. 1.0 Gbp genome and *Ilex* has a ca. 1.1 Gbp genome. We therefore project ca. 16,000 (*Solidago*) and 17,600 (*Ilex*) target fragments for these taxa, roughly corresponding to the 16,310 and 18,987 loci eventually recovered in these data sets (min_samples_locus = 4 in PyRAD; Eaton 2014). You can also digest available organelle genomes in order to determine how many non-nuclear fragments might result. In this case, *Ilex latifolia* plastid genome: 177/17 *Eco*RI/*Sph*I cuts; three of 26 of dual-enzyme fragments between 250–450 bp; three potential target fragments in the *Ilex* plastid genome. *Aster spathulifolius* (*Solidago* relative) plastid genome: 96/13 *Eco*RI/*Sph*I cuts; two of 16 of dual-enzyme fragments between 250–450 bp; therefore, two potential target fragments in the *Solidago* plastid genome.

Note: Given the natural differences in factory production of enzymes and the need to process all samples of your experiment uniformly, it is recommended that all the enzymes of a given type (e.g., restriction enzymes or T4 ligase) are of the same lot number. Estimate the total volume needed for your experiment and contact your enzyme provider beforehand.

B. Ordering oligos

Colors correspond to the “ddRAD library map” (Appendix S7), “oligo tube order” (Appendix S8), and “oligo plate order” (Appendix S9) files. Ensure that the sequences of barcodes and indices do not match enzyme pair cut sites. Note that these files assume you are using the *Eco*RI/*Sph*I enzyme pair.

1. Order the plate of 96 oligos (Fig. A1, Appendix S9). These are the 48 uniquely barcoded P1 adapters that will be ligated onto the digested fragments of each of the 48 samples in a “pool.” Each of 48 P1 adapters is made up of a “P1.1” and “P1.2” pair of oligos (rows 6 and 54 in the Appendix S9 spreadsheet is the first such pair). The P1.1 oligo contains the **Illumina sequencing primer for single end reads**, **a portion which will anneal to multiplex PCR primer 1**, a **barcode**, and the **overhang for the *Sph*I restriction enzyme**. The P1.2 oligo contains the reverse complement for both the **Illumina sequencing primer** and the **barcode**. Note that P1.2 oligos are 5′ modified with phosphate. Also note that we prefer to order the plate with each oligo frozen in Tris buffer at a normalized concentration of 200 μM in order to avoid manual elution. This format requires the (Normalization Type = Concentration Normalization, Buffer Type = Tris, and Ship Format = Frozen) ordering options on the Thermo Fisher Scientific website.

2. Order the following oligos in tubes (for oligo tube order see Appendix S8). This order comprises three components:

i. Row 6 (Appendix S8) is the single common PCR primer 1, which during PCR will add a portion where the Illumina flowcell anneals and **a portion which itself anneals to each of the 48 different P1 adapters added to the fragments in the ligation step**.

ii. Rows 7–18 (Appendix S8) are the 12 index PCR primer 2s (each with a **portion which itself anneals to the common P2 adapter** added to the fragments in the ligation step and **an index** that allows more than one pool of 48 samples to be pooled into the same Illumina lane. These primers will anneal to the common P2 adapter. If you do not need all 12 indices at this time, only prepare the ones you need. *In this case, we only prepared four of the 12. Decide which index you want to attach to which pool and* ***clearly record this detail*** *to avoid any confusion downstream.*

iii. Rows 19 and 20 (Appendix S8) are the two oligos that you will anneal to produce the single common P2 adapter. The P2.1 oligo (row 19) includes the ***Eco*RI overhang** and a section that will match a **portion of a multiplex PCR primer 2** in the amplification where you add the Illumina flowcell annealing sequences and pool indices. Note that P2.2 oligo (row 20) is 5′ modified with phosphate and 3′ modified with biotin. *The biotin modification is optional. We chose not to do the extra step of removing excess primer with specialized biotin beads as the price of the beads was unrealistic, and we removed most of the primer pairs and excess primer with our agarose gel-based size selection.*

C. DNA extraction, desalting, and quantification

All DNAs used in our pilot study were extracted with some version of the traditional CTAB protocol: see the Beck Lab 96-well protocol for an example (Beck et al., 2012). In particular, I.E.J.T. notes from experience that the use of the popular QIAGEN DNeasy extraction kits (QIAGEN, Hilden, Germany) will not routinely provide sufficient DNA for the ddRAD workflow. The CTAB method appears to provide suitable DNA quantity/quality for ddRAD, as do FastDNA kits (#MP116540600; Thermo Fisher Scientific, Waltham, Massachusetts, USA), now routinely used by one of our labs (C.T.M.). Minimum per-sample input DNA is 100 ng, with 300 ng being optimal (no more than 500 ng). The volume of per-sample DNA extract added to the digest should not exceed 10 µL, with 5 µL being optimal. Assuming a 5 µL addition, a minimum concentration of 20 ng/µL is required, with 60 ng/µL being optimal. These concentration targets were established by I.E.J.T. through trial and error. In order for the enzyme digestion to be as complete as possible, it is recommended by New England Biolabs (Ipswich, Massachusetts, USA; personal communication to I.E.J.T.), and tested by I.E.J.T. (gels available upon request), for the sample to be free of any salts. Therefore, desalting the DNA with the following protocol is recommended. This also provides an opportunity to hit the DNA concentration target and volume discussed above. Although RNA is likely not an issue, utilizing RNase during DNA extraction is advised. One can perform a post-extraction RNase/desalting as described below (Sacks Lab: https://openwetware.org/wiki/Sacks:RAD-seq#Procedure, modified by I.E.J.T. and J.B.B.).

1. Thaw (if necessary) DNAs, and briefly vortex at low speed to ensure there are no pellets.
2. Add 1 µL of RNaseA (100 µg/µL) (QIAGEN #19101).
3. Incubate at 37°C for 1 h, remove, and allow to cool to room temperature.
4. Quantify DNA concentration with a Qubit fluorometer (Thermo Fisher Scientific). Do not rely on pre-RNase Qubit values, as DNA is typically lost during this step, perhaps 20–30%.

For those samples that were already RNase-treated, begin at step #5.

5. Assuming you have 100 µL of DNA, add 100 µL 1× TE buffer to each DNA sample in a 1.7-mL tube.
6. Add 20 µL 3M sodium acetate to each DNA sample, briefly mix by *low speed* vortex.
7. Add 200 µL room temperature 100% isopropanol to each DNA sample, invert several times.
8. Place in −20°C for 1 h.
9. Centrifuge at full speed for 10 min. *Use ice or a chilled dry block to keep the tubes cold between the following steps.*
10. Pour off liquid into a beaker and save pellet.
11. Add 200 µL 70% ethanol to wash the pellet, invert a few times.
12. Spin for 1 min at max speed (just enough to pull ethanol to bottom), then pour off ethanol.
13. Spin the empty tube for 1 min at max speed, then remove ethanol by pipetting.
14. Allow pellet to dry at room temperature for several hours or overnight.
15. Resuspend in 1× TE, with TE volume based on #4 above or on a pre-desalting value if the RNase step was not performed. (60 ng/µL final concentration is the ideal).
16. Use a Qubit to confirm final concentration.
17. In order to avoid unnecessary freeze/thaw, do not freeze DNA if you are proceeding to the ddRAD preparation. Rather, store extracts at 4°C for the remainder of the experiments.

D. Adapter annealing and primer dilution

Prepare all adapters and primers you will need for the duration of the experiment. Estimate the amount of primer you need in order to dilute the proper amount. The primers are expensive, but can be used for many library preps if well taken care of. Unnecessary freeze/thaw is not recommended; therefore, do not freeze once the primers are thawed and the dilutions are made and until your library preps are ready to be sequenced. We prepared the primers below for an experiment comprising 48 samples in four pools (192 samples total). There are multiple stages of primer dilutions/incubations, so label primer plates clearly. The general process involves taking an aliquot from the purchased concentrated primer solutions, mixing the two complementary primers together in the same well, annealing them with heat, and diluting these newly annealed primers two times. Refer back to the diagram in the ddRAD library map (Appendix S7) for help.

1. Make 10× annealing buffer solution. This volume can be stored for the normal duration of buffer lifetimes.

For 100 mL:
100 mM Tris HCL pH 8.0 (10 mL of a 1M solution)
500 mM NaCl (10 mL of a 5M solution)
10 mM EDTA (2 mL of a 0.5 M solution)
Combine above in a graduated cylinder, bring to 100 mL volume with ddH_2O.

The oligos (see oligo plate order in Appendix S9) as ordered are delivered at 200 μM in the plate organized as in Fig. A1:

	1	2	3	4	5	6	7	8	9	10	11	12
A	GCAGG_SphI_P1.1	AGCTA_SphI_P1.1	ATTAC_SphI_P1.1	CTGCG_SphI_P1.1	GGATA_SphI_P1.1	TAGTA_SphI_P1.1	GCAGG_SphI_P1.2	AGCTA_SphI_P1.2	ATTAC_SphI_P1.2	CTGCG_SphI_P1.2	GGATA_SphI_P1.2	TAGTA_SphI_P1.2
B	AACCA_SphI_P1.1	ACACA_SphI_P1.1	CATAT_SphI_P1.1	CTGTC_SphI_P1.1	GGCCA_SphI_P1.1	TATAC_SphI_P1.1	AACCA_SphI_P1.2	ACACA_SphI_P1.2	CATAT_SphI_P1.2	CTGTC_SphI_P1.2	GGCCA_SphI_P1.2	TATAC_SphI_P1.2
C	CGATC_SphI_P1.1	AATTA_SphI_P1.1	CGAAT_SphI_P1.1	CTTGG_SphI_P1.1	GGCTC_SphI_P1.1	TCACG_SphI_P1.1	CGATC_SphI_P1.2	AATTA_SphI_P1.2	CGAAT_SphI_P1.2	CTTGG_SphI_P1.2	GGCTC_SphI_P1.2	TCACG_SphI_P1.2
D	TCGAT_SphI_P1.1	ACGGT_SphI_P1.1	CGGCT_SphI_P1.1	GACAC_SphI_P1.1	GTAGT_SphI_P1.1	TCAGT_SphI_P1.1	TCGAT_SphI_P1.2	ACGGT_SphI_P1.2	CGGCT_SphI_P1.2	GACAC_SphI_P1.2	GTAGT_SphI_P1.2	TCAGT_SphI_P1.2
E	TGCAT_SphI_P1.1	ACTGG_SphI_P1.1	CGGTA_SphI_P1.1	GAGAT_SphI_P1.1	GTCCG_SphI_P1.1	TCCGG_SphI_P1.1	TGCAT_SphI_P1.2	ACTGG_SphI_P1.2	CGGTA_SphI_P1.2	GAGAT_SphI_P1.2	GTCCG_SphI_P1.2	TCCGG_SphI_P1.2
F	CAACC_SphI_P1.1	ACTTC_SphI_P1.1	CGTAC_SphI_P1.1	GAGTC_SphI_P1.1	GTCGA_SphI_P1.1	TCTGC_SphI_P1.1	CAACC_SphI_P1.2	ACTTC_SphI_P1.2	CGTAC_SphI_P1.2	GAGTC_SphI_P1.2	GTCGA_SphI_P1.2	TCTGC_SphI_P1.2
G	GGTTG_SphI_P1.1	ATACG_SphI_P1.1	CGTCG_SphI_P1.1	GCCGT_SphI_P1.1	TACCG_SphI_P1.1	TGGAA_SphI_P1.1	GGTTG_SphI_P1.2	ATACG_SphI_P1.2	CGTCG_SphI_P1.2	GCCGT_SphI_P1.2	TACCG_SphI_P1.2	TGGAA_SphI_P1.2
H	AAGGA_SphI_P1.1	ATGAG_SphI_P1.1	CTGAT_SphI_P1.1	GCTGA_SphI_P1.1	TACGT_SphI_P1.1	TTACC_SphI_P1.1	AAGGA_SphI_P1.2	ATGAG_SphI_P1.2	CTGAT_SphI_P1.2	GCTGA_SphI_P1.2	TACGT_SphI_P1.2	TTACC_SphI_P1.2

Figure A1. The plate of P1 oligo pairs as delivered all at 200 μM.

2. Spin down the purchased primer plate for 10 min before lifting the foil lid. Combine an aliquot of each P1.1/P1.2 oligo pairs in the first 48 wells (A1–H6 in Fig. A2) of a standard 200 μL 96-well plate along with annealing buffer and water:

20 μL oligo P1.1
20 μL oligo P1.2
10 μL 10× annealing buffer
50 μL nuclease-free H_2O

	1	2	3	4	5	6	7	8	9	10	11	12
A	GCAGG_SphI_P1	AGCTA_SphI_P1	ATTAC_SphI_P1	CTGCG_SphI_P1	GGATA_SphI_P1	TAGTA_SphI_P1						P2
B	AACCA_SphI_P1	ACACA_SphI_P1	CATAT_SphI_P1	CTGTC_SphI_P1	GGCCA_SphI_P1	TATAC_SphI_P1						P2
C	CGATC_SphI_P1	AATTA_SphI_P1	CGAAT_SphI_P1	CTTGG_SphI_P1	GGCTC_SphI_P1	TCACG_SphI_P1						
D	TCGAT_SphI_P1	ACGGT_SphI_P1	CGGCT_SphI_P1	GACAC_SphI_P1	GTAGT_SphI_P1	TCAGT_SphI_P1						
E	TGCAT_SphI_P1	ACTGG_SphI_P1	CGGTA_SphI_P1	GAGAT_SphI_P1	GTCCG_SphI_P1	TCCGG_SphI_P1						
F	CAACC_SphI_P1	ACTTC_SphI_P1	CGTAC_SphI_P1	GAGTC_SphI_P1	GTCGA_SphI_P1	TCTGC_SphI_P1						
G	GGTTG_SphI_P1	ATACG_SphI_P1	CGTCG_SphI_P1	GCCGT_SphI_P1	TACCG_SphI_P1	TGGAA_SphI_P1						
H	AAGGA_SphI_P1	ATGAG_SphI_P1	CTGAT_SphI_P1	GCTGA_SphI_P1	TACGT_SphI_P1	TTACC_SphI_P1						

Figure A2. The "annealing plate" of 48 P1 adapter oligo pairs (wells A1–H6) and the single P2 common adapter split across wells A12 and B12. All are now at a 40 μM stock solution.

3. Similarly, combine the P2.1 and P2.2 oligos, annealing buffer, and water in one 1.7-mL tube:

60 μL oligo P2.1
60 μL oligo P2.2
30 μL 10× annealing buffer
150 μL nuclease-free H_2O

4. Mix, then split the tube contents into wells A12 and B12 (Fig. A2), each containing 150 μL.

5. Cover this annealing plate with a silicone mat (#1223S33; Axygen via Thomas Scientific, Swedesboro, New Jersey, USA) and run the adapter annealing cycling protocol. Peterson et al. (2012) note the following conditions "97.5° C for 2.5 minutes, and then cool at a rate of not greater than 3° per minute until the solution reaches a temperature of 21°C." Three degrees per minute is 0.05°C/second, but all the cyclers we have investigated can only cool as slowly as 0.1°C/second. We therefore wrote a protocol that started at 97.5°C for 2.5 min, then cooled slowly 3 degrees at a time by lowering at the 0.1°C/second rate for 30 seconds (down 3 degrees) and then holding at that new cooler temperature for 2 min. This repeats until 21°C is reached, which is held for 10 min. Following annealing, seal the plate well with tight-fitting strip tube caps or plastic/foil, as the silicone mat is not made for storage. Once these primers are annealed, you can store them at 4°C for the duration of the experiment. Following annealing, you now have a 40 μM stock solution of each of the 48 P1 adapters and the P2 adapter in the 96-well "annealing plate" (Fig. A2).

6. Make a 4 µM solution of these 48 P1 adapters in another 96-well plate. This dilution is made with a 1× solution of the 10× annealing buffer solution detailed above.

For 50 µL of 4 µM P1 adapter (in a new 96-well plate):
5 µL of 40 µM stock annealed P1 adapters per well
45 µL 1× annealing buffer per well

7. Remove the P2 common adapter from the A12 and B12 positions in the annealing plate and keep as a 40 µM stock solution in a 1.7-mL tube.

8. Make a 4 µM solution of the P2 adapter in another 1.7-mL tube.

For 200 µL of 4 µM P2 adapter:
20 µL of 40 µM stock annealed P2 adapters
180 µL 1× annealing buffer

9. Before the annealed P1 and P2 adapters are ligated to the digested DNAs they need to be diluted to a custom "working" concentration based on average DNA concentration of samples in that pool, the cut frequency of both enzymes from in silico digests, and volume of adapters needed. You have already diluted them to 4 µM in the step above in a plate (P1 adapters) and single tube (P2 adapter). A new plate and new tube are then *custom diluted on a per-pool basis* based on the ligation molarity calculation spreadsheet provided by Peterson et al. (2012).

In our case, we are using cut frequency values from in silico digests, so we would use the corresponding Peterson et al. in silico spreadsheet: https://docs.google.com/spreadsheets/d/1fcg6mYESNEvi8jfu3T1Bbue-rsVqgeC6RIXjzT9CRrc/edit?hl=en_US&hl=en_US#gid=0. In this "ddRAD ligation molarity calculator" (Fig. A3), you need only change the values in the green-shaded boxes. In reality, you will likely only change the values for "Initial DNA mass (µg)" and "cut frequency (bp)." Initial DNA mass is simply the average DNA mass for that pool (in µg) that will go into the ligation. We have calculated this in two ways: the less labor-intensive way is simply to calculate the mean DNA mass *initially added to the digestion*. The more labor-intensive way is to do a Qubit quantification of all samples *after the bead cleanup of the double digestion* and calculate the post-digestion mean DNA mass. Because some DNA will inevitably be lost during digestion (ca. 50% in some cases), this post-digestion mean will provide a more accurate measure of the amount of DNA actually going into the ligation. We have successfully produced and sequenced libraries using both measures. A final option is to simply assume this ca. 50% loss and adjust your initial DNA mass accordingly. Cut frequency is simply the average distance between cut sites for each restriction enzyme. We interpret this as the average size of fragments resulting from single-enzyme in silico digests of the best available genomic sequence you can obtain. Geneious will typically present this summary value in the "statistics" (%) tab following digestion. Alternatively, the data can be exported and the mean fragment size calculated manually. In *Daucus*, the average fragment size was approximately 6500 bp for the *Sph*I digestion and 3500 bp for the *Eco*RI digestion. Because *Sph*I creates the overhang onto which the P1 adapter ligates, this value (6500) is entered for "PI." Because *Eco*RI creates the overhang onto which the P2 adapter ligates, 3500 is entered for "P2." It is also important to note that (based on a miscommunication), we used values that did not correspond to these calculations for the *Solidago* and *Ilex* pools. Instead we used 18,666 and 6800 for P1 and P2, respectively. Given that we nevertheless observed considerable success for these two pools, *and* that the values used for the *Boechera* and *Draba* pools here have been successfully used in distantly related genera (I.E.J.T., personal communication), we are not certain how critical it is to optimize them. We did not experiment with "target adapter fold excess" or "target adapter volume/rxn (µL)" and kept those at default values. "Annealed adapter conc (pmol/µL)" will remain at 4 if you have diluted your adapters to 4 µM in the step above as recommended. "Volume of working stock to make (µL)" is set at 200, and 200 µL worked well for us. For our *Boechera* pool, based on these Peterson calculations we added 9.09 µL of each 4 µM P1 adapter to 190.91 µL 1× annealing buffer in a new "working P1 *Boechera* plate" AND 30.3 µL of 4 µM P2 adapter to 169.70 µL 1× annealing buffer in a new "working P2 *Boechera* tube." You will create a different working P1 plate and working P2 tube for each species pool.

Boechera			Input appropriate values in green shaded fields
Initial DNA mass (ug)	0.24		mass of double-digested genomic DNA in ligation
	P1	P2	
cut frequency (bp)	10000	3000	average distance between sites for each enzyme (from genome
fragment mass (g/mole)	6600000	1980000	
sample mass (g)	2.40E-07	2.40E-07	
fragments/sample (moles)	3.64E-14	1.21E-13	
ends/sample (moles)	7.27E-14	2.42E-13	
ends/sample (pmoles)	0.07	0.24	
target adapter fold excess	5	5	molar excess of adapter to genomic DNA ends
target adapter/sample (pmoles)	0.36	1.21	
target adapter volume/rxn (ul)	2	2	desired amount of working adapter stock to add to ligation rxn
adapter working conc (pmol/ul)	0.18	0.61	
annealed adapter conc (pmol/ul)	4	4	concentration of annealed adapter stock
fold dilution to working stock	22.00	6.60	
volume of working stock to make (ul)	200.	200.	desired volume of working adapter stock
**annealed adapter stock (ul)**	**9.09**	**30.30**	
**1x annealing buffer (ul)**	**190.91**	**169.70**	
**working stock to add to ligation (ul)**	**2**	**2**	

Figure A3. Example of a ligation molarity calculator used for creating the *Boechera* adapter working solutions.

At this point, you have the following sets of oligos/adapters at various concentrations:

A. The original plate of P1 and P2 oligos as delivered at 200 μM (Fig. A1). This should be stored at −20 °C for long term, but kept at 4°C until the protocol is finished.
B. The "annealing plate" of 48 P1 adapters at 40 μM (Fig. A2). This should also be stored at −20 °C for the long term, but kept at 4°C until the protocol is finished.
C. A plate of 48 P1 adapters at 4 μM. This should be stored at 4°C until the protocol is finished.
D. A set of plates of the 48 P1 adapters of "working solutions" for each pool. Store at 4°C until the protocol is finished.

E. Double digest

1. Prepare a spreadsheet noting the position of your DNAs in your plates. It is helpful to have the tubes with your genomic DNAs in a 96-well rack (#HS2345A; Heathrow Scientific from Thomas Scientific) to facilitate the transfer.

2. Add 5 μL of genomic DNA (100–500 ng total, 300 ng optimal) from each sample to the corresponding well in a new 96-well plate (#MPS-500; Phenix Research Products, Candler, North Carolina, USA). If not immediately moving on to the digestion, add a foil or plastic seal (Thermo Fisher Scientific #14-222-342 or equivalent) to your plates to prevent evaporation. However, it is advised to make the digestion plate the same day as the planned digestion. Otherwise, cover with a sterilized silicone mat (Thomas Scientific/Axygen #1223S33), place the plates on ice, and proceed with the digestion master mix preparation. Your 5 μL DNA volume will evaporate or condense on the cover very quickly when on ice, so if time has passed, be sure to spin down the plate again before adding the enzyme mixture.

3. Prepare fresh double digestion master mix (MM) on ice in 5-mL vials (#1505-1000; USA Scientific, Ocala, Florida, USA). Add water/buffer first, briefly vortex/spin each enzyme before slowly pipetting and adding them, and vortex/spin the total MM between enzyme additions. Note that the following calculations are based on 192 samples, calculated for 200 samples to accommodate liquid loss.

Component	μL/rxn × # wells	=	MM μL
restriction enzyme 1 (SphI-HF, New England Biolabs product #R3182L)	0.75 μL × 200	=	150 μL
restriction enzyme 2 (EcoRI-HF, New England Biolabs product #R3101S)	0.75 μL × 200	=	150 μL
10× CutSmart buffer (supplied by NEB with enzymes)	2.50 μL × 200	=	500 μL
nuclease-free H_2O	16.0 μL × 200	=	3200 μL
raw DNA ~200–300 ng	5.0 μL × n/a	=	n/a

Note: If you have low DNA quantities (>100 ng), you can modify the above recipe in order to add more DNA in place of nuclease-free water. This mix comprises ca. 15 U of enzyme per reaction, and you can increase enzyme addition (~20–25 U per reaction) if you observe incomplete digestion during size selection.

4. Add 20 µL of this MM to each 5 µL of genomic DNA in the plates (on ice). Seal each plate with sterilized silicone mats, “bump vortex” each plate very briefly, then briefly spin plates to settle contents.

5. With silicone mats still in place, wrap the plates tightly with aluminum foil and place in a 37.5°C incubator for 3 h. Alternatively, you can place them in a thermocycler with only the silicone mat. If you have an orbital shaker that can sit inside the incubator, use it on a low orbital setting. Although the silicone mats expand in the heat and seal the wells nicely, you can place weights (e.g., dry blocks) on the plates to keep the mat in place to ensure they stay seated on the plate. Move plates to 4°C if not proceeding to the bead cleanup step immediately, but do not freeze. In either case, just before proceeding to the bead cleanup step, allow the digestion plates to come to room temperature for the beads to work properly. Once at room temperature, spin the plates for 10 min at maximum speed to draw down any condensation before removing the silicone mat.

Note: Wash the silicone mats thoroughly with a 30% Alconox solution, rinse three times with water, three times with distilled water, then three times with nano-pure water. Wrap the mats in foil and autoclave for the next use.

F. Bead cleanup of double digest in 96-well plates

Note: Although AMPure beads are recommended by Peterson et al. (2012), we have successfully used SeraMag beads (#65152105050250; GE Healthcare, Little Chalfont, Buckinghamshire, United Kingdom) with a “homebrew” buffer shared in our common wet lab (Evolutionary Genomics Lab, Department of Integrative Biology, University of California, Berkeley). Mag-Bind RxnPure Plus beads (#M1386-01; Omega Bio-Tek, Guangzhou, China) are another economical option. Store beads at 4°C in a 50-mL conical vial (“falcon tube”) wrapped in foil as the solution is slightly light sensitive, and vortex vigorously before each use. Figure A4 shows some of the items you will need.

1. Prepare fresh 70% ethanol and calculate the volume of beads you will need:

per sample:
1.5 × sample (1.5 × 25 µL digest volume = 37.5 µL beads)
per plate:
(96 × 37.5 µL = 3600 µL beads)

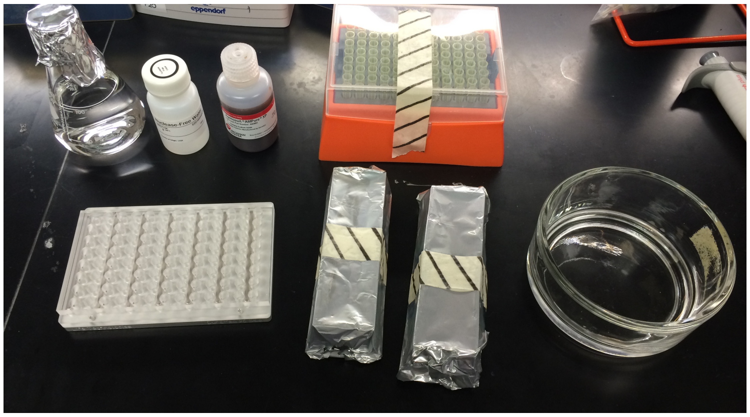

Figure A4. Some of the items you will need for bead cleanups: 70% ethanol, beads, nuclease-free water, tips, plates, reservoirs, dish for waste.

2. Pipette the needed volume of beads into a reservoir. Add 37.5 µL beads to each well with a P200 multichannel pipette and mix by slowly pipetting 10 times. Be careful not to to introduce bubbles. Note that in our experience the bead solution adheres to pipette interior and it is therefore difficult to completely eject 100% of the solution. Therefore, when you eject the tip, a small amount of bead/digestion solution is frequently deposited on the pipette shaft tip. If this happens, clean with ethanol and Kimwipes or perhaps use filter tips.

3. Incubate for 5 min. While waiting, pop any major bubbles in the wells with a new individual pipette tip. At this point, your plate will look like Fig. A5 (left panel).

4. Place plate on plate magnet (Life Technologies DynaMag -96 Side Skirted #12027) and wait 15 min (solution will clear relatively quickly). At this point, your plate will look like Fig. A5 (right panel).

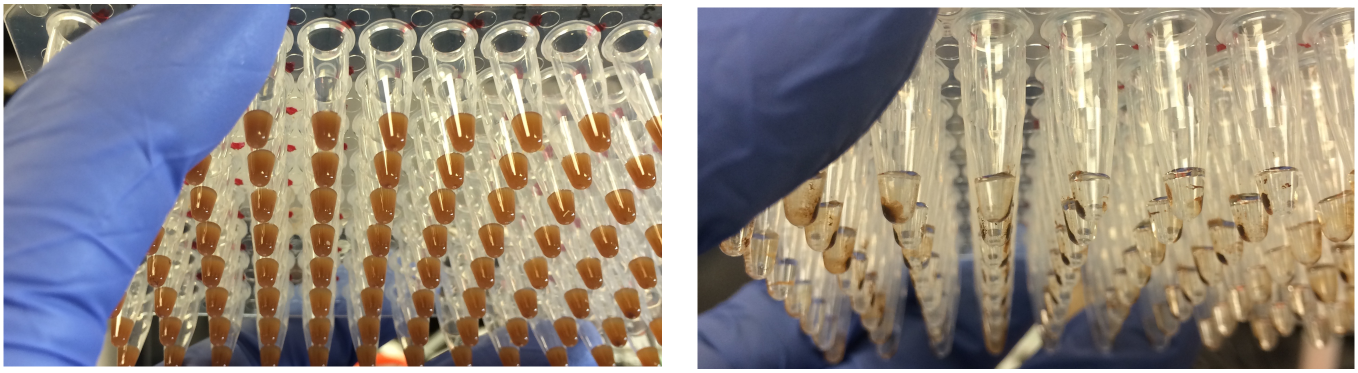

Figure A5. Your plate following bead addition (left) and after 15 min on the magnet (right).

5. Keep the plate on the magnet. Remove all but 5 µL of the clear solution (ca. 58 µL, Fig. A5 right panel) from the wells with a P200 multichannel pipette. Take care not to disturb the pellet by taking advantage of the position of the pellets on one side of the wells. Discard liquid waste into a glass dish.

6. Add 200 µL of 70% ethanol and wait 1 min. Pull off ethanol.

7. Add another 200 µL of 70% ethanol and wait 1 min. Pull off ethanol and follow with a P10 pipette to remove the last residual ethanol drop. Allow plate to dry until pellets begin to visibly crack (≥30 min). A hand lens works well for evaluating pellet dryness.

8. Remove plate from magnet. Add 30 µL of nuclease-free H_2O and pipette over the pellet many times until the pellet is in suspension. Some will not fully rehydrate; work on these by pipetting up and down with a single-channel pipette.

9. Return plate to the magnet, allow pellet to reform, and remove 30 µL of liquid (now with eluted and cleaned DNA) into a new, labeled "digested, cleaned DNA" 96-well plate. Seal with foil (Thermo Fisher Scientific #14-222-342 or equivalent) and store at 4°C if not used immediately. Again, do not freeze.

G. Adapter ligation

1. Program a thermocycler for the ligation reaction. The following parameters are from Peterson et al. (2012):

23°C for 30 min (room temperature incubation)
65°C for 10 min (heat kill)
cool at 2°C per 90 seconds until you reach room temperature, which is a rate of ca. 0.02°/second

As before, our cycler could not cool that slowly, so we wrote a protocol that began at 23°C for 30 min, heat killed at 65°C, then cooled slowly *3 degrees at a time* by lowering at the 0.1°/second rate for 30 seconds (down 3 degrees) and then holding at that new cooler temperature for 2 min. This was repeated until room temperature (23°C) was reached.

2. Prepare a master mix of all components except the P1 adapters and DNA in a 1.7-mL tube. Briefly vortex and spin down the T4 DNA ligase and ligation buffer before adding to master mix.

**Ligation component**	**Per reaction**	**Master mix for a pool of 48 samples (×50)**
P2 common adapter (also custom "working solution")	2 µL	100 µL
10× ligation buffer (provided by New England Biolabs)	4 µL	200 µL
T4 DNA ligase (New England Biolabs #M0202L)	2 µL	100 µL
Nuclease-free H_2O	10 µL	500 µL

3. Add 18 µL of this master mix and 2 µL of the “working solution” P1 adapter to the 30 µL DNA in each well of the “digested, cleaned DNA” plate. Add the P1 adapter with a multichannel pipette, but add the master mix with a single-channel pipette. Add a clean silicone mat, vortex/spin down the plate, and run the Peterson et al. (2012) adapter ligation protocol in a thermocycler as detailed above.

H. Bead cleanup of each pool in 2-mL tubes

1. Prepare fresh 70% ethanol.

2. For each pool, combine the 48 samples into one 5-mL tube (a separate tube for each pool), gently mix by pipetting, then equally aliquot the contents into three round-bottomed 2.0-mL tubes (USA Scientific #1620-2720). These 2.0-mL tubes will fit in the magnet rack and accommodate both the ligated DNA and beads (Fig. A6). Record the total collective volume of each pool.

3. Add 1.5× volume beads to each tube, mix by pipetting, and wait 5 min.

4. Apply tubes to a magnetic separation rack. New England Biolabs sells six-tube (#S1506S) or 12-tube versions (#S1509S). Ensure the position of the tube allows the developing pellet to form on the side of the tube with some tube space below (Fig. A6). A homemade spacer can be inserted below the tube cap or tube tip if needed.

5. Wait 15 min for the solution to clear.

6. While the samples are still on the magnet, remove the clear solution with a pipette. Note that at times the sample/bead mixture will unexpectedly appear “fuzzy” and not fully adhere to the magnet. Proceed as normal as the beads tend to adhere fully to the magnet during the following ethanol steps.

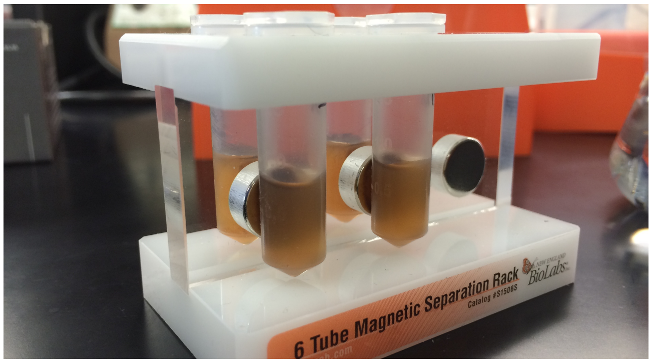

Figure A6. Beads (with digested, adapter-ligated fragments attached) moving toward the magnet. Solution will clear more with time.

7. Add 1 mL of 70% ethanol well above the pellet and wait 1 min. It is OK if the pellet is somewhat disturbed during the ethanol addition, as the beads will migrate back to the magnet.

8. Remove the ethanol with a pipette, add another 1 mL of 70% ethanol and wait 1 min.

9. Pipette off all ethanol (go back with a small volume pipette to remove as much as you can) and allow the pellet to air dry with the tubes open.

10. When the pellet is visibly dry, remove the tube from the magnet and use 60 µL of nuclease-free water to wash the pellet off the tube side. Pipette above the pellet so the water flows over the pellet. Repeat by drawing up the volume and re-applying until the pellet is completely in solution.

11. Once in solution, incubate the sample at room temperature for 5 min to allow the DNA to disassociate from the beads and go into solution in the water.

12. Re-apply the tube to the magnet and wait for the solution to clear.

13. Withdraw the 60+ µL of water per tube into a *single* low-retention microfuge tube (#14-222-171; VWR, Radnor, Pennsylvania, USA) for each pool. This is 180 µL per pool if you used three tubes per pool.

14. Bead-clean the 180 µL volume of each pool *a second time*, repeating steps 3–13 of part H above, except elute in 40 µL of nuclease-free water in step 13. This reduced volume will allow a pool to be run in a single gel well during size selection.

I. Size selection

Size selection involves targeting digested, ligated fragments of a size range suitable for the type of Illumina sequencing you are planning. The method below is known as the "freeze and squeeze" method, an economical alternative to automated size selection with a Pippin Prep (Sage Science, Beverly, Massachusetts, USA).

1. Prepare test gels to determine the gel volume necessary to accommodate the 40 µL pool in your lab's gel rig.

2. Clean the gel rig/comb before size selection. It does not need to be sterile, simply washed thoroughly to avoid introducing additional DNA fragments.

3. Prepare a 2% agarose gel of the required volume. We recommend adding gel stain (Phenix Research Products #RGB 4103) (~0.005% in the agarose gel) to the hot agarose directly before pouring. This eliminates the need to stain the gel in a potentially contaminated post-electrophoresis stain bath.

4. Remove the comb once the gel cools. Add 15 µL 5× loading buffer (#BIO-37045; BioLine, Memphis, Tennessee, USA) and 5 µL 100% glycerol (pipette very slowly) to each 40 µL pool, mix by pipetting, then load the entire pool contents into a single gel lane. The added glycerol will ensure the sample sinks into the well. Run 10 µL of BioLine 100 bp HyperLadder (Phenix Research Products #DNAL-1000-100) in alternating wells. Load both the sample and ladder slowly, making sure you allow time for the sample to settle. A six-well gel would be arranged as follows, with ladder on each side of each sample:

well 1	well 2	well 3	well 4	well 5	well 6
ladder	**pool 1**	ladder	ladder	**pool 2**	ladder

5. Run at 60 V. Check after 15 min to ensure the sample has entered the gel and the dye front is moving in a straight line. After 30 min, remove the gel tray/gel and briefly check band separation on a UV box. Run until the 100, 200, 300, 400, and 500 bp bands of the ladder are clearly visible (Fig. A7). While the gel is running, prepare a small plastic disposable Petri dish (Thermo Fisher Scientific #FB0875713A) and clean razor blade for each pool.

Note: At this point you will know if your digestion was successful. If your gel looks like Fig. A7 (left), the digestion was likely too incomplete to continue. The middle and right panels of Fig. A7 illustrate more complete digestions, with a long smear of DNA with very little remaining near the well, and an insignificant amount at the bottom of the gel. If your digestion appears incomplete, it would be wise to start over, adding more units of enzyme to the digestion step, desalting DNA (if you skipped this step), or using more starting DNA.

6. When you are satisfied with band separation, place the gel box on a UV box and take a pre-cut image.

7. With a clean razor blade, cut between ca. 250 and 350 bp (if targeting fragments ca. 300 bp). It is important to cut straight up and down. Remove the gel plug and transfer to a clean, labeled Petri dish.

Note: This is a somewhat imprecise process, dependent on the quality of the ladder and your visual interpretation of the gel. Fragment analysis of our subsequent pools demonstrates that this 250–350-bp cut resulted in a slightly larger fragment size window, ca. 200–400 bp. We highly recommend asking your chosen sequencing core to perform a fragment analysis prior to sequencing in order to inform your choice of sequencing approach.

8. Take a second picture to document the size range you have removed.

9. Freeze the plugs at –20°C until frozen solid. Often this means simply freezing overnight.

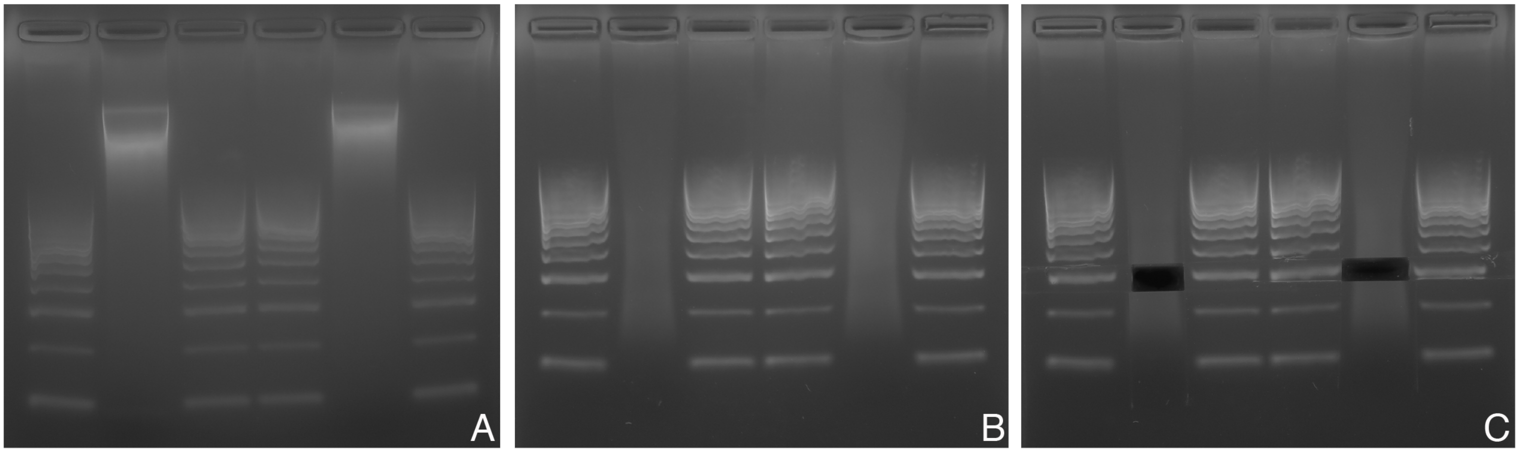

Figure A7. Size selection. Gel images of pools flanked by BioLine 100 bp HyperLadder (bottom band is 100 bp, increasing by 100 bp toward the well). (A) Two pools with incomplete digestion. (B) Pre-cut image of two pools with successful digestion. (C) Post-cut image of panel B.

10. With (newly) gloved hands, jam each frozen plug into a SpinX column (VWR #29442-756). The plugs are often too large; if so, use a new blade and a clean glass dish to carefully cut them into smaller pieces. If the plug is too large for one SpinX column, divide the plug into two columns. This can get a bit messy as the plug thaws, so clean the outside of the column tubes and change gloves between samples.

11. Spin at 13,000 rpm for 30 min at room temperature.

12. Rinse any remaining DNA from the agarose by adding 100 µL of nuclease-free water to each tube above the agarose and spinning at 15,000 rpm for 10 min or until the agarose is dry.

J. Bead cleanup and quantification of each size-selected pool

1. Transfer the contents of each SpinX column to a round-bottomed 2.0-mL tube (USA Scientific 1620-2720), noting the starting liquid volume of each pool. These volumes are often between 600–700 µL, so a single pool and (1.5× volume) beads can likely be accommodated in a single 2-mL tube.

2. Clean each pool as described in section H above, using a final elution volume of 60 µL per pool.

3. Quantify the DNA concentration of each pool elution with a Qubit DNA High Sensitivity kit (Thermo Fisher Scientific #Q32854). Pool concentrations are now typically 5–10 ng/µL.

K. Phusion PCR of cleaned, size-selected pools

We recommend the Phusion High-Fidelity PCR Kit (New England Biolabs #E0553L). Each Phusion reaction requires a maximum of 20 ng DNA, and multiple individual reactions can therefore be performed per pool: (Qubit concentration × 60 µL volume per pool) / 20 ng per PCR = reactions possible per pool. Note that some pooled DNAs might need to be diluted to 2 ng/µL if they exceed this concentration, as you add 10 µL of DNA to each reaction; 20 ng per PCR is the maximum input suggested by Phusion. Some pools may have concentrations < 2.0 ng/µL. In these cases, one can add more pool aliquot to each reaction (e.g., 20 µL per reaction instead of 10 µL).

Note: Before you try to amplify the entire pool in one run, we recommend testing amplification with one or two aliquots of each pool to ensure amplification is possible. Adjustments to annealing temperatures and cycle numbers may be needed.

1. Dilute PCR primer 1 (“PCR1,” row 6 in the oligo tube order file [Appendix S8]) and the multiplex/index PCR primer 2s (rows 7–18 in that file) from 200 μM to 10 μM. The number of multiplex PCR primer 2s you will need to dilute is dependent on the number of pools you are working with (you need one index per pool). Record the index choice per pool.

2. Prepare the following Phusion PCR master mix:

PCR component	Per reaction	Final concentration	Master mix for strip of eight samples
5× HF buffer (supplied)	4.0 μL	1×	32.0 μL
dNTP (10 mM) (supplied)	0.4 μL	200 μM	3.2 μL
DMSO (100%) (supplied)	0.6 μL	3%	4.8 μL
PCR primer 1 (10 μM)	2.0 μL	0.5 μM	16.0 μL
DNA Poly Phusion	1.0 μL	0.1 U/μL	8.0 μL

3. For each reaction, add a 10-μL aliquot of pool DNA and 2 μL of the appropriate 10 μM multiplex/index PCR primer 2 to a 0.2-mL tube.

4. Add 8 μL of the Phusion master mix to each tube. Vortex/spin the master mix before the addition and the PCR tubes following the addition. Total volume of typical reactions is 20 μL, but the volume may be greater if the pool DNAs are <2.0 ng/μL.

5. Run this program:

Phusion PCR program:
1) 98°C for 30 s
2) 98°C for 10 s
3) 79°C for 30 s. This annealing temperature is based the suggested PCR protocol from Peterson et al. (2012) and trial/error by I.E.J.T.
4) 72°C for 30 s
5) GOTO step 2 (29×) (the number of cycles was tested numerous times by I.E.J.T.)
6) 72°C for 10 min
7) 4°C hold
8) END

Note that the number of cycles for next-generation sequencing is generally kept low (8 to 12 cycles). I.E.J.T. found that for this procedure, 8 to 12 cycles were simply not enough to sufficiently amplify the library. The increased number of cycles did not affect the sequencing quality.

L. Library quantification, bead cleanup, final quantification, and storage

1. Following PCR, combine the multiple reactions from each pool and analyze each total library using Qubit. Library concentrations should be ca. 20–40× the pre-PCR pool DNA input concentration if the amplification was successful.

2. If so, bead-clean each library as in section H above, using final elution volumes based on pre-clean Qubit values:

if pre-clean Qubit is >100 ng/μL, elute in 90 μL
if pre-clean Qubit is 40–100 ng/μL, elute in 60 μL
if pre-clean Qubit is <40 ng/μL, elute in 40 μL

3. Qubit following bead cleanup and report this concentration and volume to your sequencing facility. Your library should have a concentration of 30–70 ng/μL. If you checked the concentration with the BR Qubit kit and did not detect DNA, try again with the HS kit. Even with low concentrations, sequencing might still be successful.

4. Store the final library at 4°C for a short time period, or −20°C if it cannot be sequenced quickly.

5. Prior to sequencing, your sequencing facility should perform a fragment analysis on the library (e.g., Bioanalyzer, TapeStation [Agilent Technologies, Santa Clara, California, USA]). If large amounts of adapter/primer dimers are observed, these can be removed with a lower bead : DNA ratio (0.8×) cleanup or a Pippin size selection. This is typically done for a minimal fee at the sequencing facility (Fig. A8).

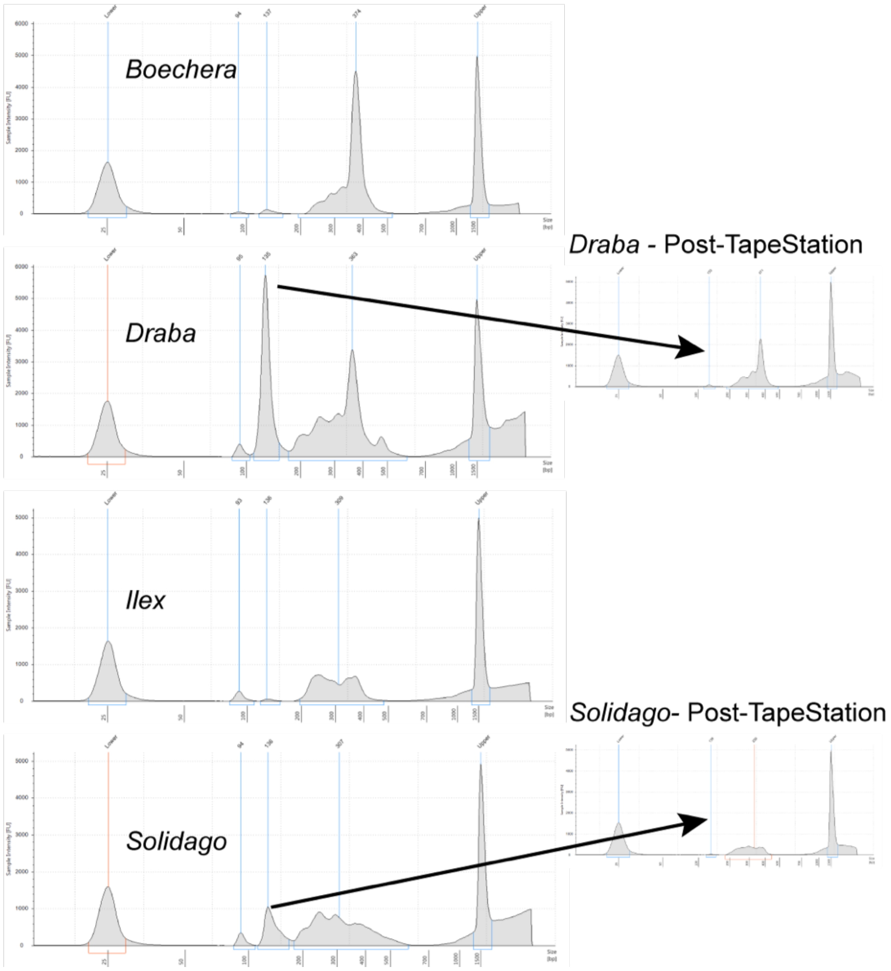

Figure A8. Library fragment analysis. The ca. 25-bp and ca. 1500-bp peaks are size standards. The amplified library ranged from ~200–350 bp in each pool. The peaks at ca. 95 bp and ca. 135 bp were viewed as likely adaper/primer dimer and were removed with a Pippin prep size selection.

Recommended schedule

1) DNA extraction, desalting, quantification = week 1
2) Primer dilution and adapter annealing = week 2, day 1
3) Preparation of digestion plate = week 2, day 2
4) Double digest = week 2, day 2
5) Bead cleanup of digested DNA = week 2, day 3
6) Adapter ligation = week 2, day 3 or 4
7) Bead cleanup of digested, ligated, pooled DNA = week 2, day 5
8) Size selection (gel electrophoresis) = week 2, day 6
9) Size selection (freeze and squeeze) = week 2, day 7

10) Bead cleanup of size-selected pooled DNA= week 2, day 7
11) Phusion PCR, Qubit quantification, and subsequent bead cleanup = week 3, day 1

Specialized reagent/equipment list

Note that some standard lab equipment and reagents are not listed here.

Geneious (www.geneious.com)
Plate of oligos (Invitrogen via Thermo Fisher Scientific)
Tube of oligos (Invitrogen via Thermo Fisher Scientific)
RNaseA (100 μg/μL) (QIAGEN 19101)
Qubit fluorometer (Invitrogen via Thermo Fisher Scientific)
Qubit dsDNA BR assay kit (Invitrogen via Thermo Fisher Scientific Q32850)
Qubit dsDNA HS assay kit (Invitrogen via Thermo Fisher Scientific Q32851)
96-well rack for 2-mL tubes with lids (Heathrow Scientific from Thomas Scientific, HS2345A)
96-well plates (Phenix Research Products MPS-500)
foil seals (Axygen via Thermo Fisher Scientific 14-222-342)
reusable silicone mat for 96-well plates (Axygen via Thomas Scientific 1223S33)
*Sph*I-HF (New England Biolabs R3182L) (if you choose this enzyme pair)
*Eco*RI-HF (New England Biolabs R3101S) (if you choose this enzyme pair)
10× CutSmart buffer (supplied by New England Biolabs with enzymes)
5-mL vials (USA Scientific 1505-1000)
SeraMag beads (GE Healthcare 65152105050250) OR Mag-Bind RxnPure Plus (Omega Bio-Tek M1386-01)
50-mL conical vials (Thermo Fisher Scientific 14-432-22)
T4 DNA ligase (New England Biolabs M0202L)
2.0-mL tubes (USA Scientific 1620-2720)
magnetic separation rack for 1.5-mL tubes: (New England Biolabs S1506S)
DynaMag magnetic separation rack for 96-well plates (Invitrogen via Thermo Fisher Scientific 12-027)
low-retention microfuge tubes (VWR 14-222-171)
GelRed stain (Phenix Research Products RGB 4103)
5× blue loading buffer (BioLine BIO-37045)
DNA ladder (Phenix Research Products DNAL-1000-100)
disposable Petri dishes (Thermo Fisher Scientific FB0875713A)
SpinX columns (VWR 29442-756)
Phusion High-Fidelity PCR KIT (New England Biolabs E0553L)

LITERATURE CITED

Beck, J. B., P. J. Alexander, L. Allphin, I. A. Al-Shehbaz, C. Rushworth, C. D. Bailey, and M. D. Windham. 2012. Does hybridization drive the transition to asexuality in diploid *Boechera*? *Evolution* 66: 985–995.

Eaton, D. A. 2014. PyRAD: Assembly of de novo RADseq loci for phylogenetic analyses. *Bioinformatics* 30: 1844–1849.

Peterson, B. K., J. N. Weber, E. H. Kay, H. S. Fisher, and H. E. Hoekstra. 2012. Double digest RADseq: An inexpensive method for de novo SNP discovery and genotyping in model and non-model species. *PLoS ONE* 7: e37135.
