# Supplementary material for: A basic ddRADseq two‐enzyme protocol performs well with herbarium and silica‐dried tissues across four genera
Source: Appl Plant Sci. 2020 Apr 23;8(4):e11344. doi: 10.1002/aps3.11344 (PMC7186894; doi:10.1002/aps3.11344)

DOI

APPENDIX S5. Unrooted phylogeny of 40 *Solidago* samples using a data set filtered at the min_samples_locus = 4 threshold. Bold branches denote those with >95% maximum likelihood bootstrap support. For sample names, refer to Appendix S2.

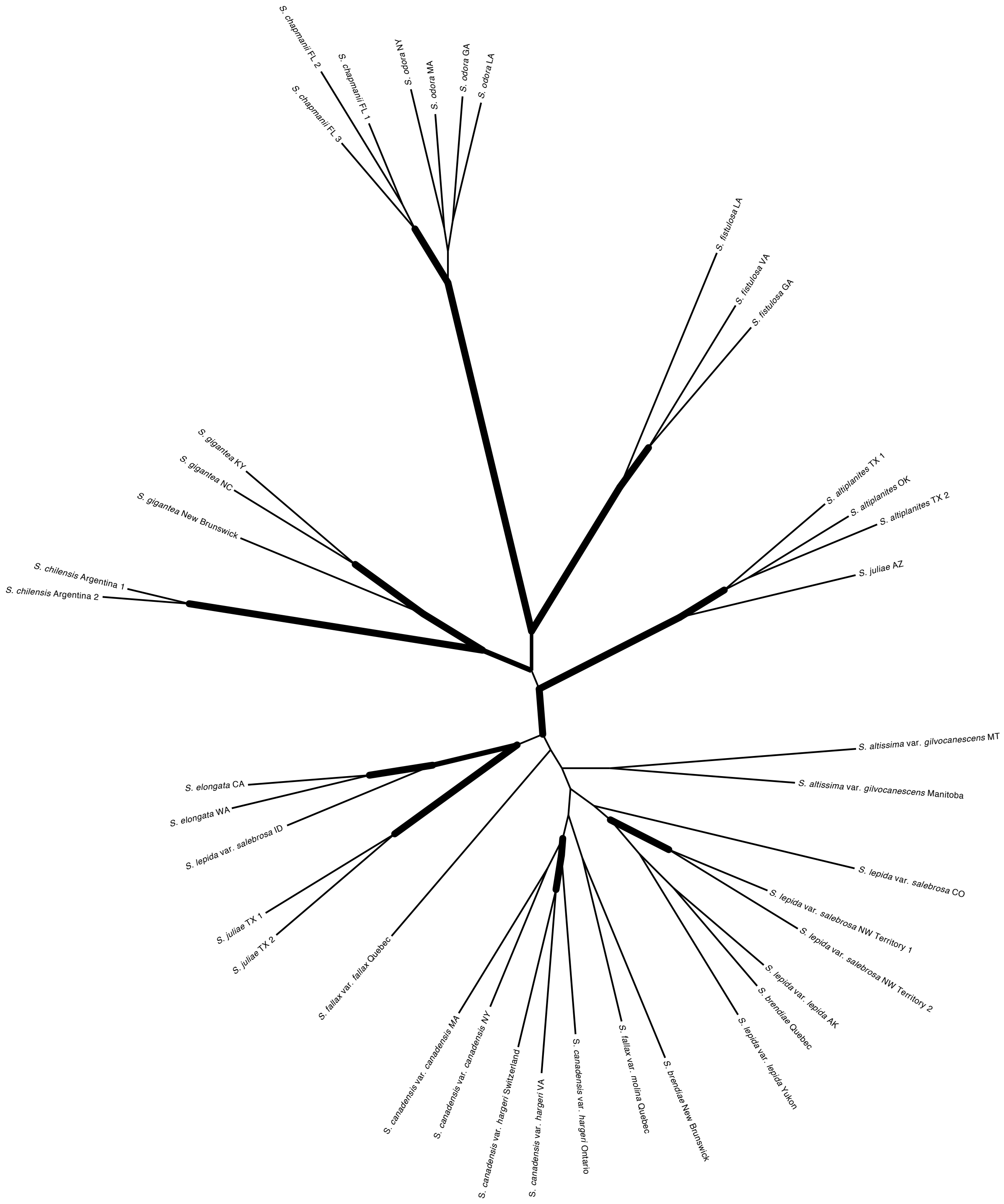

Supplement: Supplementary file 5 — APPENDIX S5. Unrooted phylogeny of 40 Solidago samples using a data set filtered at the threshold min_samples_locus = 4. [file APS3-8-e11344-s005.pdf]
