# Supplementary material for: A basic ddRADseq two‐enzyme protocol performs well with herbarium and silica‐dried tissues across four genera
Source: Appl Plant Sci. 2020 Apr 23;8(4):e11344. doi: 10.1002/aps3.11344 (PMC7186894; doi:10.1002/aps3.11344)
Supplement: Supplementary file 7 — APPENDIX S7. ddRAD library map, with colors corresponding to Appendix S1. [file APS3-8-e11344-s007.docx]

Jordon-Thaden et al.—Applications in Plant Sciences 2020 8(4)—Data Supplement S7

DOI

Appendix S7. ddRAD library map, with colors corresponding to Appendix S1.

**(1) DNA digested with SphI (left) and EcoRI (right)**

**5’ NNNNN 3’**

**3’ GTACNNNNNTTAA 5’**

**(2) Adapters annealed to each other, with one of 48 PI adapter shown on the left and the common P2 adapter shown on the right**

**5’ (P1.1) ACTCTTTCCCTACACGACGCTCTTCCGATCTGCATGCATG AATTAGATCGGAAGAGCGAGAACAA\3Biotin 3’ (P2.2)**

**3’ (P1.2) TGAGAAAGGGATGTGCTGCGAGAAGGCTAGACGTAC TCTAGCCTTCTCGTGTGCAGACTTGAGGTCAGTG 5’ (P2.1)**

**(3) Target DNA ligated to the P1 and P2 adapters, which will be pooled and size selected**

**5’ ACTCTTTCCCTACACGACGCTCTTCCGATCTGCATGCATGNNNNNAATTAGATCGGAAGAGCGAGAACAA\3Biotin 3’**

**3’ TGAGAAAGGGATGTGCTGCGAGAAGGCTAGACGTACGTACNNNNNTTAATCTAGCCTTCTCGTGTGCAGACTTGAGGTCAGTG 5’**

**(4) Add the PCR 1 (forward primer) and PCR2 (reverse primer), after samples have been through size selection**

**5’ AATGATACGGCGACCACCGAGATCTACACTCTTTCCCTACACGACG (PCR1 -->)**

**5’ ACTCTTTCCCTACACGACGCTCTTCCGATCTGCATGCATGNNNNNAATTAGATCGGAAGAGCGAGAACAA 3’**

**3’ TGAGAAAGGGATGTGCTGCGAGAAGGCTAGACGTACGTACNNNNNTTAATCTAGCCTTCTCGTGTGCAGACTTGAGGTCAGTG**

(<--PCR2) **CGTGTGCAGACTTGAGGTCAGTGTAGTGCTAGAGCATACGGCAGAAGACGAAC 5’**

Final Library (with PCR1 and PCR2 illustrated)

**5’ AATGATACGGCGACCACCGAGATCTACACTCTTTCCCTACACGACG (PCR1)**

**5’ AATGATACGGCGACCACCGAGATCTACACTCTTTCCCTACACGACGCTCTTCCGATCTGCATGCATGNNNNNAATTAGATCGGAAGAGCACACGTCTGAACTCCAGTCACATCACGATCTCGTATGCCGTCTTCTGCTTG 3’**

**3’ TTACTATGCCGCTGGTGGCACTAGATGTGAGAAAGGGATGTGCTGCGAGAAGGCTAGACGTACGTACNNNNNTTAATCTAGCCTTCTCGTGTGCAGACTTGAGGTCAGTGTAGTGCTAGAGCATACGGCAGAAGACGAAC**

(PCR2) **CGTGTGCAGACTTGAGGTCAGTGTAGTGCTAGAGCATACGGCAGAAGACGAAC 5’**

Final Library that will be sequenced

**5’ AATGATACGGCGACCACCGAGATCTACACTCTTTCCCTACACGACGCTCTTCCGATCTGCATGCATGNNNNNAATTAGATCGGAAGAGCACACGTCTGAACTCCAGTCACATCACGATCTCGTATGCCGTCTTCTGCTTG 3’**

**3’ TTACTATGCCGCTGGTGGCACTAGATGTGAGAAAGGGATGTGCTGCGAGAAGGCTAGACGTACGTACNNNNNTTAATCTAGCCTTCTCGTGTGCAGACTTGAGGTCAGTGTAGTGCTAGAGCATACGGCAGAAGACGAAC**

**67 bp to the left of the target sequence (all minus the NNNNs), 68 bp to the right of the target sequence.**

**Total library should be between 200 and 300 bp**

**Pink = barcode (1 of 48)**

**Blue = enzyme hang sequence**

**Red = digested DNA sample with target sequence**

**Green = once forked adapter that permitted initial PCR cycle only from P1 end, and after the PCR2, it is the sequencing primer for paired-end reads**

**Orange and Grey highlighted = flowcell annealing from PCR 1**

**Brick red = sequencing primer for single-end reads**

**Brown = index (1 of 12)**

**Ocean = primer for multiplex reads**

**Green highlighted = PCR binding sites**
